# Sex, Sexuality, and Intimate Relationships Among Afghan Women and Men of Refugee Background Living in Melbourne, Australia: Experiences, Opportunities, and Transcultural Tensions

**DOI:** 10.1007/s10508-022-02296-6

**Published:** 2022-09-23

**Authors:** Alana Russo, Belinda Lewis, Razia Ali, Atiq Abed, Grant Russell, Stanley Luchters

**Affiliations:** 1 Burnet Institute, 85 Commercial Road, Melbourne, VIC 3004 Australia; 2 School of Public Health and Preventive Medicine, Monash University, Melbourne, VIC Australia; 3 Refugee Health Program, Monash Health Community, Monash Health, Dandenong, VIC Australia; 4 School of Primary Health Care, Monash University, McMahons Road, Frankston, VIC Australia; 5 Melbourne, VIC Australia; 6 Southern Academic Primary Care Research Unit, Department of General Practice, Monash University, Notting Hill, Australia; 7 Department of Population Health, Aga Khan University, Nairobi, Kenya; 8 International Centre for Reproductive Health, Department of Public Health and Primary Care, Ghent University, Ghent, Belgium

**Keywords:** Refugee, Migrant, Sex, Culture, Australia

## Abstract

Over the last two decades, Afghanistan has been a leading country of origin for asylum seekers and refugees arriving in Australia. It is widely recognized that humanitarian migrants experience poorer sexual and reproductive health than the broader population. In turn, a body of research has emerged exploring the sexual and reproductive health of the local Afghan community. However, this has predominantly focused on youth or perinatal experiences, and less attention has been given to the broader relational and social dimensions of sexuality. Accordingly, this research aimed to explore the perspectives and experiences of married Afghan women and men as they navigate and negotiate sex, sexuality, and intimate relationships following settlement in Melbourne, Australia. A total of 57 Afghan women and men participated in six focus group discussions and 20 semi-structured interviews. Male participants described the ways that having increased access to sex and sexually explicit materials in Australia is creating opportunities for them to establish more fulfilling sex lives. Many women also described a growing awareness of sexuality, although often expressed difficulty prioritizing and claiming more pleasurable sexual encounters for themselves. However, concerns about sexual freedom are also creating new challenges for the Afghan community living in Australia in relation to sex and relationships. For example, men expressed fears about women exercising sexual liberties outside of the home, and this appeared to place women’s everyday behavior under increased scrutiny. Women also voiced concerns about how easily men can access sex outside of marriage within Australia, and described how this amplified their sense of obligation to be sexually compliant and meet their husband’s desires. This study provides new insights into the ways that Afghan community members are moving between societies, and how their understandings of sexual participation, pleasure, desire, health, consent, and capacity for self-determination are being challenged, reshaped, and reconstructed throughout this process.

## Introduction

Sexual health is defined as a holistic state of wellbeing that includes physical health, as well as mental, emotional, and social wellbeing related to sexuality (WHO, 2020). Therefore, sexual health is not only concerned with fertility regulation, the prevention and treatment of sexually transmissible infections, and maternal healthcare, but also with the pursuit of positive and respectful intimate relationships, and safe, pleasurable, and fulfilling sexual experiences (WHO, 2020). Sexuality is a central aspect of humanity, and as such, sexual health is integral to the overall health and wellbeing of individuals, couples, families, and the wider contexts of society (Hibler & Colombini, 2002; WHO, 2020).

In many settings, migrant communities experience poorer sexual and reproductive health than the broader population, including increased rates of sexually transmissible infections, unplanned pregnancies, and obstetric complications (McMichael, 2013; Metusela et al., 2017; Svensson et al., 2017). Despite migrant communities’ apparent need for sexual and reproductive healthcare, these groups generally have low participation within mainstream services (Botfield et al., 2016; Gagnon et al., 2002; Mengesha et al., 2017). This has been attributed to a range of barriers to access, such as varying levels of proficiency in the language of the host country, challenges navigating unfamiliar healthcare systems, financial hardship, and competing settlement priorities (Hach, 2012; Svensson et al., 2017). Further, many migrant communities come from cultures where the subject of sexuality is highly sensitive, and cloaked in shame and silence (Botfield et al., 2016; Rogers & Earnest, 2014; Svensson et al., 2017; Ussher et al., 2017). Inhibited discussion of sexuality has been found to constrain sexual and reproductive health knowledge and sexual health-seeking behaviors among some migrant groups (Hach, 2012; McMichael & Gifford, 2009a, 2009b; Ussher et al., 2012).

In addition, social and cultural factors—such as religion, gender roles, and familial and social expectations—are central to guiding and shaping definitions, beliefs, meanings, and practices around sex and intimate relationships (Dawson & Gifford, 2001; Dworkin et al., 2007; Hach, 2012). This has particular implications for migrant communities, as they are often required to negotiate contrasting, contending, and competing sociocultural norms around gender, power, and pleasure (Hach, 2012; Hawkey et al., 2018, 2019; Hondagneu-Sotelo & Hondagneu-Sotelo, 1994; Khoei et al., 2008; Muchoki, 2015). The intersections and interactions of ideals and conventions contribute layers of complexity to relationships and sexual and reproductive health decision-making, while also creating opportunities for new sexual cultures to emerge (Ahmadi, 2003; Kalra & Bhugra, 2010).

People of refugee background are often considered a distinct group within the broader migrant population. This is due to heightened health and social vulnerabilities associated with the refugee experience, which often includes: forced displacement; torture and trauma; abrupt cultural dislocation; loss, separation, and fragmentation of family and community; extensive and hazardous journeys in search of safety; and extended periods with limited access to formal education and health services (Agier, 2008). As such, people of refugee background generally experience poorer health than other migrant groups and the wider population, and this includes an excessive burden of sexual and reproductive health need (Janssens et al., 2005).

Over the last two decades, Australia has accepted between 13,000 and 20,000 people of refugee background annually (Phillips, 2017). Throughout this period, Afghanistan has consistently been a leading country of origin for humanitarian arrivals (Australian Government, 2019). Many social, cultural, and political factors associated with Afghanistan starkly contrast the experiences of Australian-born people. For example, traditional Afghan society is collectivist, heavily patriarchal, and practices gender segregation; thereby constructing strong cultural expectations around the roles of women and men (Kandiyoti, 2005; Rostami-Povey & Poya, 2007). Additionally, Islamic beliefs are widespread throughout Afghanistan, and religiosity has been found to further play into gendered power relations, and underpin the acceptability of various sexual practices and behaviors (Coleman & Testa, 2008; Rostami-Povey & Poya, 2007; Wray et al., 2014). Therefore, Afghanistan-born people^1^ living in Australia and comparable settings are likely to have unique sexual and reproductive health experiences and needs.

Recent sexual and reproductive health research involving the Afghan community living in Australia has included inquiry into health meanings, beliefs, knowledge, and barriers to sexual health service access among youth and unmarried young adults (McMichael & Gifford, 2009a; Wray et al., 2014). Additionally, there is a body of research prioritizing married women and men, which is predominantly focused on perinatal experiences and family planning (Russo et al., 2015, 2019; Shafiei et al., 2012; Yelland et al., 2016). These studies have provided valuable insights into the complex tensions that people from Afghanistan experience around sexual and reproductive health, and the ways that these impact on their physical and emotional health, relationships, and family wellbeing. However, there has been less focus on the relational and social dimensions of sexuality; such as desire, satisfaction, pleasure, and identity, and the ways that rapid cultural transitions influence individuals and couples within these intimate domains. Establishing greater knowledge in this area has the potential to inform the development of services and programs that foster healthy sexuality, and illuminate footholds that can be utilized by Afghan people themselves to initiate social change within their communities. Accordingly, this research aimed to explore the perspectives and experiences of married Afghan women and men as they navigate and negotiate sex, sexuality, and intimate relationships following settlement in Melbourne, Australia.

## Method

### Participants

The most recent national census data in Australia stated that there were 46,800 Afghanistan-born residents (ABS, 2018b). Settlement often occurs in clusters, and therefore, the local impact of these arrivals has been significant in certain regions. For example, the City of Greater Dandenong and the neighboring City of Casey, located in the southeastern region of Melbourne, are home to the largest community of Afghanistan-born people within the state of Victoria. In 2016, it was reported that 14,617 Afghanistan-born people live within these two municipalities alone; and this constitutes over 30% of all Afghanistan-born people within Australia (ABS, 2018a). The current study was undertaken within these catchments.

To be included this study, participants needed to be between 18 and 49 years of age, married, born in Afghanistan, of refugee background, and currently living in Melbourne. In alignment with recommendations from community researchers, Afghan women and men who arrived in Australia more than 15 years ago were excluded from this research. This was due to an interest in exploring cultural transitions, which may be less apparent over a settlement period longer than this. People unable to communicate in either Dari (the most widely-spoken language by Afghanistan-born people) or English were also unable to participate due to practicalities related to the bilingual support and translated materials that were available.

Participants were recruited using a purposive, maximum variation sampling strategy. This approach enabled the deliberate selection of individuals with the knowledge and experience required to fulfill the research objectives (Creswell & Clark, 2011; Patton, 2002). Community researchers led the recruitment process, which involved approaching existing contacts within the Afghan community, and asking them to help identify other individuals meeting the inclusion criteria. During recruitment, community researchers provided all participants with a verbal explanation of the aim of the research project and what involvement entailed. All participants then received an Explanatory Statement and Consent Form, which was available in Dari and English, and also offered verbally in both languages to overcome varying literacy proficiencies. All participants provided verbal and written consent, and completed a short sociodemographic questionnaire, prior to data collection. This process occurred until all sections of the sampling frame were fulfilled, with the final number of participants guided by the point at which data saturation was achieved.

### Procedure

This qualitative study was comprised of two phases; Phase 1 involved six focus group discussions, and Phase 2 involved 20 semi-structured interviews. Qualitative methods were considered appropriate for this research, as they are flexible and well suited to exploring complex issues among vulnerable groups (Creswell & Clark, 2011; Liamputtong, 2013). The two-phased approach, in which focus group discussions were conducted prior to the semi-structured interviews, was particularly fitting in respect the exploratory and sensitive nature of this research. The focus groups facilitated lively interaction between participants, and were an efficient way to capture multiple perspectives (Baum, 2008). This method was appropriate to establish broad priority themes, however, achieving depth of individual stories can be challenging within the group context (Baum, 2008). ‘Group think’ is a further limitation of focus group discussions, in which participants may be reluctant to express perspectives and experiences that contrast with the majority of attendees (MacDougall & Baum, 1997). Accordingly, Phase 2 complimented the focus group discussions by utilizing semi-structured interviews conducted in a private setting to explore the lived experiences of individual participants in greater depth and detail (Baum, 2008). This was particularly valuable for exploring subject areas of heightened sensitivity, such as relationships, sex, and cultural change.

In alignment with a community-based participatory research approach, community researchers from the priority population were recruited and trained to work collaboratively with the academic team throughout each stage of the research process (Minkler & Wallerstein, 2010). Consistent with the literature, the high level of community involvement, including shared and equal project ownership, was intended to: optimize cultural competency throughout the research process; nurture trust and rapport between the research team and the participants; overcome logistical challenges related to community access and language barriers; and, ultimately, improve data quality and enhance rigor (Minkler & Wallerstein, 2010).

The foundations of this study draw on intersectionality and contemporary cultural studies. First, intersectionality was applied throughout data collection, organization, and analysis, and offered a framework to explore the multiple marginalities of participants, relating to gender, culture, religion, socioeconomic status, and migrancy (Carastathis, 2014). Contemporary cultural studies provided an additional conceptual lens to consider cultural meanings and practices surrounding health and sexuality as fluid, dynamic, and constantly open to change, rather than being unitary, static, or fixed (Lewis & Lewis, 2014). This article presents a selection of cross-cutting themes exploring the complex and changing nature of identities, beliefs, and practices. Therefore, contemporary cultural studies was the primary theory framing this paper.

### Measures

Data collection involved two distinct phases; the focus group discussions were conducted and analyzed prior to commencing the interviews. All six focus group discussions were gender-specific, and facilitated in Dari by a male or female community researcher accordingly. It was important to conduct the focus group discussions with women and men separately due to practices of gender segregation which are common within traditional Afghan society, and furthermore, in consideration of the sensitive nature of the subject area. Community researchers were accompanied by another member of the research team, who attended focus group discussions to provide logistical support, undertake observational note-taking, and lead formal debriefing with the community researcher at the end of the session. Focus group discussions were structured around a set participatory activities. This involved using fictional narratives, sorting cards, and image boards to generate discussion around family planning, contraception, pregnancy, sex, relationships, and health seeking. Prompt questions were developed in close consideration of the theoretical frameworks and embedded within the activities. This ensured that central issues of gender, culture, religion, cultural transition, and meaning making within the context of sex and relationships were thoroughly explored and discussed. All focus group discussions were conducted at a community health center frequented by Afghan women, men and families, and were up to two and a half hours in duration.

Following the focus group discussions, 20 semi-structured interviews were undertaken. The interview guide used for these was informed by the focus group discussion findings and the research objectives. The interview guide was comprised of seven priority themes, including: Family size ideals; Contraception: beliefs, experience, and practice; Decision-making; Pregnancy intention; Roles and relationships; Sex, affection, and intimacy; and Health seeking and resources. A series of non-prescriptive, open-ended prompt questions were linked to each theme. The interview tool was refined through a piloting period, and regularly reviewed throughout data collection.

Participants had the option of having their interview conducted in Dari or English. This resulted in seven interviews being conducted in English, and 13 interviews being conducted in Dari. Interviews were an average of one and a half hours in duration. All participants received an A$40 gift voucher to recognize their time contribution, and cover any travel expenses incurred.

Focus group discussions and interviews were digitally recorded to allow for subsequent reference and transcription. Data collected in English were transcribed verbatim. For the large quantity of data collected in Dari, translation and transcription was a rigorous process. This began with community researchers listening to recordings several times to familiarize themselves with their content. Then, community researchers sat with another member of the research team to work through the recording, which was interpreted by the community researcher, and transcribed directly into English. This collaborative process enabled any areas of ambiguity to be earmarked, questioned, and clarified. Community researchers then carefully reviewed each transcript while listening to the recording, in order to correct any errors or missing details. A second party also transcribed a proportion of the data to enable cross-checking and determine consistency. The original and re-transcribed documents were examined and the variation between them was considered minimal; therefore this process was deemed to be valid and appropriate. Minimal grammatical corrections were made to the transcripts by a native English-speaking member of the research team to improve coherence and flow.

All data were de-identified by replacing participants’ names with a unique numerical code and pseudonym. The research team had sole access to any identified or re-identifiable data. All forms, recordings, and transcripts were managed in accordance with ethical guidelines.

### Data Analysis

Initial explorations of the data included reviewing field and debriefing notes, re-reading transcripts, and reflective memoing (Rubin & Rubin, 2005). This was followed by a series of coding cycles, which employed a combination of data driven coding and concept driven coding (Gibbs, 2007). Codes were consistently reviewed and revised throughout the data collection process, until data saturation was determined. A proportion of transcripts were independently coded by members of the research team to assess inter-rater reliability (Mays & Pope, 1995). There was a high rate of agreement between the coded transcripts, at approximately 85%.

Codes were then organized into a nested framework, which was agreed upon by the research team (Gibbs, 2007). Initial concepts and themes were presented to the community researchers for their input and further refinement. Data were rearranged within thematic groups, and findings were compared and contrasted with existing literature (Ritchie & Lewis, 2003). Computer assisted qualitative data analysis software NVivo QSR International was used to code, organize, and retrieve data.

Respondent validation was central to ensuring that the community was involved in all stages of the research process. Therefore, at the end of each focus group discussion and interview, researchers reviewed their notes, and then provided participants with a summary of the discussion and invited them to add and clarify details as necessary. In addition, at the time of data collection, all participants were verbally offered the opportunity to provide feedback on the research findings at the completion of data collection by making contact with the research team. However, due to a limited response, a subgroup of participants was directly contacted by phone and invited to attend an interactive session to hear about the findings that emerged, and reflect on whether these were considered representative of the community’s opinions and experiences. This process enhanced rigor and established confirmability (Mays & Pope, 1995).

This study was approved by Monash University Human Research Ethics Committee, Project Number CF16/1777-2016000917.

## Results

A total of 57 Afghan women and men participated in this study. Each participant could only take part in one data collection activity. Eighteen women took part in one of three female-only focus groups, and 10 women participated in interviews. Nineteen men took part in one of three male-only focus groups, and 10 men participated in interviews. Women were between the ages of 23–43 years with a mean age of 33.6 years. Men were between the ages of 22–49 years with a mean age of 35.5 years. While all participants were born in Afghanistan, only eight were living there directly prior to arriving in Australia. Before arriving in Australia, 29 participants were living in Pakistan, 15 were living in Iran, four were living in Indonesia, and one was living in the United Arab Emirates. Participants had been living in Australia for between four months and 14 years, with a mean length of time in Australia of 5.5 years.

Five key themes have been selected for inclusion in this paper based on their contribution of new knowledge to the subject area. These are: Engagement with new sexual cultures and content; Information and health seeking; Understandings and practices related to sex; Women’s desire and sexual satisfaction, and; Initiating, accepting, and declining sex.

### Engagement with New Sexual Cultures and Content

Participants, particularly males, described their experiences of observing and engaging with new sexual cultures and content since arriving in Australia. These participants broadly viewed Australian culture as being sexually liberal; with sexual content, resources, and activities described as being widely acceptable and easily accessible. This was frequently contrasted with, what participants perceived to be, the more covert and restricted nature of sexuality within Afghanistan and neighboring countries, such as Pakistan and Iran. Media, including commercial advertising, television, and movies—both mainstream and pornographic, emerged as predominant sources of exposure to new sexual cultures and content. In addition, some men spoke about their experiences of engaging with sex workers, particularly before they were married, or throughout periods of family separation; that is, when they had arrived in Australia but their wives were still overseas awaiting visa approval.

I’m here for five years [without my wife, who is in Afghanistan]…So whenever I need [sex], I will go somewhere and empty myself. I’m in Australia, if I want sex, I go somewhere and do sex. I go to brothel, pay $200 or $400 I will spend. Reza, man, early 30s, 5 years in Australia (interview participant).

Participants of both genders frequently implied that sex is fundamentally a man’s need. Within this, a number of male respondents explained that they felt it was important for men to have safe options to release their sexual tension to prevent inappropriate sexual acts, such as sexual harassment and assault, within the community. For these participants, brothels provided an outlet to meet their sexual “needs.” This option was particularly suitable for some men in the study, as they described that practices of gender segregation, which are commonplace within Afghan society, have contributed to their lack of experience and confidence to meet and socialize with women in everyday social settings.

Men are changing now [in Australia]…In Pakistan it's different—even if people are 30 years old, if they are not married, they are still virgins. If they want to find [a brothel] they can, but if the police catch you, there will be trouble. But here, everything is legal, it’s no problem, free country…I think this is very good for myself...If you [don’t go to brothels], maybe you go and do something wrong…especially in summer time [with girls wearing] good dresses…Because people need [sex], it's like food…Sometimes you watch something on Facebook, on Internet, and get horny. If you go [to a brothel], afterwards you relax. If you don't go there, so what you do after? For sure you do something wrong (sexual harassment or assault). Especially myself, I’ve never been in a bar…I feel shy, I can't go and talk with girls…So, I go pay my money, that's a good option for me. Abdul, man, early 30s, 6 years in Australia (interview participant).

While many men viewed their exposure to, and engagement with, new sexual cultures positively, some women and several men intimated concerns about how this might adversely impact marital relationships. For women, fears often related to the ways that men’s sexual experiences in Australia may shift the sexual expectations they have of their wives.

[Afghan men] were not exposed to those things before, and now they like pornography, they like having access to brothels...Sometimes they will go and watch these things, and then they will be expecting their wives to do the same thing, but their wife might not be able to do. I think that’s a huge, huge problem, it weakens [the relationship… Nafeesa, woman, late 20s, 10 years in Australia (interview participant).

For men, fears centered on the shifting power dynamics between genders. Within this, several men expressed concerns that Australian culture allowed Afghan women to have multiple sexual partners.

Men have no value here…Why do they say if women want to sex everyone, they can? I mean, if women want to do sex, she can access easily here. She can do anything. I feel this culture is very bad for men, but it is good for women, because here, women are gaining control. Mohammad, man, late 30s, 7 years in Australia (interview participant).

This appeared to be contributing to a sense of impeded freedom for some women, who described that they felt their own everyday behavior was under increased scrutiny since living in Australia.

I will tell you about my husband. When I was in [home country], I usually go out from morning and come home in the evening. I visited many people, and he never knew where I was. But here, every day he needs to know my whole life; what I did, what I eat, what I wear, when I showered. Yalda, woman, mid 20s, 2 years in Australia (focus group participant).

### Information and Health Seeking

Doctors emerged as the most highly trusted and valued source of sexual health information and assistance, particularly for men. Doctors were described as “*mahram*”—approved by God within their profession, exempt from cultural practices of gender segregation, and permitted to engage in discussions of personal nature. Some men spoke openly about how living in Australia had changed their understandings of optimal sexual health and performance, and increased their awareness of the treatments that are available to achieve this. Participants frequently contrasted this with their experience of living in Afghanistan, in which they felt they had limited access to sexual health knowledge, resources, and services.

[Doctors] have education…they know about everything. We can go to the doctor for the things that we cannot control ourselves. For example, during sex I have this condition…I quickly ejaculate and I wanted to get better so I enjoy my sex more. Only the doctor will sort out this issue. In Australia we can go, it is not uncomfortable. But in Afghanistan we feel shy to speak to doctor or to anyone about this problem. Sultan, man, early 20s, 2 years in Australia (focus group participant).

Some participants also described using books and the Internet to learn about sex and related issues. These participants highly valued the privacy inherent within this approach.

If you have access to Internet, it’s an easy way. Because whatever you want to know, whatever questions you have, you can get it. I have some shyness to ask from doctor. Zackariah, man, early 30s, 6 years in Australia. (focus group participant).

I wasn't very aware about intercourse. To be honest, I thought maybe the first time when you do intercourse, the girl is going to get pregnant. So I did a bit of Google…and after that I became more aware. Mustafa, man, late 20s, 10 years in Australia (interview participant).

Overall, participants expressed a desire to move away from the cultural silencing of sexuality, and appreciated that in Australia they had a greater degree of freedom to seek health information from a range of sources to address sexual health needs. Many women and men expressed their hopes that living in Australia would ensure that their children would ultimately be well informed about relationships and sexual health.

I really don’t want our children to face the difficulties that we faced. Difficulties related to sex, related to everything. We need to give our children information on the issues we had…so that they won’t have to face the same problems. Batool, woman, mid 30s, 2.5 years in Australia (focus group participant).

### Understandings and Practices Related to Sex

Many participants indicated that within Afghan culture, sex is frequently constructed and practiced as a perfunctory, male-centric activity. Participants described that this was often incontestably accepted and unchallenged as the cultural norm within Afghanistan and neighboring regions.

My mother-in-law said, “Men, you know, they don't care. They just put their pants down and they shoot and then they just leave.” So I think it's stuck in my mind, it's just men doing their part and leaving, and then for women to clean up…There is not a lot of foreplay in Afghan culture...they don't want to do it…They are like, “Oh, yep, that's it, we're done!” I'm not too sure if it’s because of religion. I think it's more just the culture. Aquila, woman, early 30s, 12 years in Australia (interview participant).

However, a many men spoke openly about the ways that their understandings of sex had shifted and evolved since living in Australia. This most frequently included increasing recognition of women as equal and valued partners within sexual encounters. At times participants associated these new learnings with gaining a deeper understanding of intimacy, pleasure, and fulfillment, and they were generally optimistic and enthusiastic about progressing sexual knowledge and practices within their community.

In 10 years of marriage…I didn’t know women also have a peak; they need to reach at that level…When I came here [I] see in movies and I learn more. And when my family came here, slowly I fixed it…I always had wrong thinking related to sexual relationship with women. I thought that when we do sex, only I am important. But now I understand that not only I need to be satisfied, my wife needs to be satisfied as well…We need to give this information to our community; to men from our culture. Tajj, man, mid 30s, 9 years in Australia (interview participant).

Afghani culture related to sex is very bad. Because in the past women do not enjoy from their sex life, they do not understand what sex is. And a lot of men come and say Afghani women have complained about sex [in the past], but they come in this country and now they understand orgasms, and they now understand what life is, what sex in Australia is. It is big and positive change for them. Mohammad, man, late 30s, 7 years in Australia (interview participant).

For some participants, the move towards more mutual, intimate, and pleasurable sexual experiences included incorporating oral sex. These participants described that this practice was unfamiliar to them prior to arriving in Australia and engaging in sexual activities with women outside of their cultural community. While some men were open to incorporating oral sex into their sex lives, other participants indicated that this conflicted with their religious beliefs.

…the way Western people do sex we are not allowed. Because with the mouth we pray, we speak to our mother and father, and we eat. They will use it for sex…According to our beliefs, it’s not clean; it’s a sin. Ali, man, late 30s, 4 years in Australia (focus group participant).

Mate, how we have sex [in Australia] is totally different...For example, in Afghanistan or in Pakistan, no one (neither men nor women) knows about sucking (oral sex)…I said it directly, don’t get upset. In those counties, sex is about 5 or 7 minutes…You just lift it up, spit on your hand, rub it on her… push it in, and then it’s finished…And you don’t understand the enjoyment of sex and life…But here, you take off her clothes gently, and she takes off your clothes…Here you give her enjoyment (perform oral sex) for 5 minutes…and you do sex slowly, you do sex very relaxed. But over there, these things do not exist. The wife doesn't know how to [perform oral sex], and the men don’t know [either]…In Afghanistan, I didn't enjoy [sex] properly. I didn't ask from my wife, “Are you satisfied with this sex or not?” She didn’t scream [and] now I understand that she didn't enjoy much…I was a donkey before, I feel it now. But if I go [back to Afghanistan] and be with my wife, I will do sex in a better way to satisfy her. Reza, man, early 30s, 5 years in Australia (interview participant).

### Women’s Pleasure and Sexual Satisfaction

Many female participants described that since living in Australia, they had become more aware of the potential for women to gain pleasure from sexual activity. For some women, sexual satisfaction was openly discussed within their marriages. Several female respondents indicated that they felt comfortable expressing their personal sexual preferences to their partners to achieve more mutually enjoyable sexual encounters.

He always asks, “You are happy from this? You feel happy or satisfied this way?” And if I say, “No, do this way”, he always listen. Jamila, woman, early 30s, 2 years in Australia (interview participant).

However, most women talked about positive sexual outcomes as seeming unattainable. These women indicated a lack of knowledge around alternative ways to achieve sexual pleasure beyond intercourse, and at times, described that they felt very little agency around their own pleasure. Some women intimated indifference as to the importance of whether or not they attained satisfying sexual experiences for themselves. Rather, this group of female participants described that it was their husbands who were motivated and proactive in encouraging women’s sexual release, by initiating conversations and suggesting health-seeking opportunities through popular culture and more formal health resources.

People have told me that women can have the same thing like a man. Believe me, now I have three children, but I have no experience of this; I have not had that sort of feeling…I can see him, when he finishes he feels so relaxed...But I did not feel anything…My husband tells me that I am cool, and he also tells me to look in the movies, and it is this sort of feeling…He always tries to help me, and to control himself until I have an orgasm. But I always say, “Do it, finish your work. How long you wait for me?” Saera, woman, early 30s, 9 years in Australia (interview participant).

I don't know if my body’s different, but I don't get much pleasure from intercourse. My husband, he does…We started researching [on the internet] about this and there is a percentage of women and it's normal for them not to have any pleasure…I could be one of them. Ah, there are so many other things that I like in life… so it doesn't really matter…(participant laughing) It's so funny, he has tried so much, poor guy. Sometimes I feel so sorry for him so I say “No, I liked it!”, and he's like, “You don't have to lie to me.” He told me to talk to my GP about it and see what’s happening…Sometimes he thinks that something's wrong with him because he cannot make me feel satisfied. Nafeesa, woman, late 20s, 10 years in Australia (interview participant).

However, several female participants described that they continued to experience great difficulties in speaking openly and comfortably about sexual issues with their partners, and this was a hurdle for them in addressing their lack of sexual satisfaction.

Sometimes women feel uncomfortable during sex. They do not feel happy and they do not enjoy to the degree they should enjoy, they don’t. And I can’t say to anyone, and even to husband, because I feel embarrassed. Safia, woman, early 30s, 6 months in Australia (focus group participant).

In 18 years, it's just a few times that I enjoyed sex. When [my husband] tries to ask me about it...I just say, “I don't know! I don't have any ideas about it!” Feroza, woman, mid 30s, 12 years in Australia (interview participant).

### Initiating, Accepting, and Declining Sex

Participants described the varying ways that they negotiate sex within their marriages. The vast majority of women, across all age groups, explained that they never initiated sex with their husbands. Some women attributed this to a lack of desire or yearning to engage in sexual activity. For others, the idea of initiating sex sat uncomfortably beyond their roles as Afghan women, and was tied to feelings of inhibition and embarrassment. Additionally, several women suggested that initiating sex was connected to a power-play between themselves and their husbands.

I can't say “Come, I want sex.” All Afghani women feel shy to say…it's our habit…We don't want to feel less (needy or inferior) in front of our men; if he wants sex he needs to come to us and say! Ghulsom, woman, early 30s, 4 years in Australia (interview participant).

Paradoxically, most women said they were reluctant to directly refuse sex when their husbands approached them. On the one hand, when men ask for sex, women feel a powerful sense of holding control over something that men want; and on the other hand, a sense of powerlessness to have agency over sexual decision-making. The reoccurring notion that sex is a fundamental need for men underpinned some women’s explanations for this. For others, the mandate to fulfill their husband’s sexual requests was linked to their religious beliefs; although other female participants contested this connection.

Every second night [my husband] wants sex, and if he asks, I will not argue with him. Because he is a man, he needs sex more than me. Anything he wants I need to do it…My religion says even if I am praying and my husband asks for sex, if I leave my prayer and I fulfill his desire it is not a sin. This is my religion. And, if the nights your husband asks you for the sex and you pretend you are asleep and you ignore, the angels will curse you all night until morning because you did not fulfill your husband’s needs… Zamira, woman, late 30s, 4 months in Australia (interview participant).

There are some people that are saying, “Oh yes, [wives] have to do sex because of religion”…But I'm saying, in religion it is not mentioned that you have to if you don’t want…A lot of people twist religion. Aquila, woman, early 30s, 12 years in Australia (interview participant).

Additionally, several women illuminated the ways that living in Australia, and the perceived accessibility of sex within this societal context, compounded their sense of obligation to meet their husband’s sexual needs. For these women, sexual compliance was, in part, fueled by the fear of living in a more sexually liberal society.

Sometimes I agree on having sex, but I don’t want to do it…I do it, but sometimes I’m not happy…If I say no, he is man, he will go after another [woman]…In Australia it’s free culture; anyone can build sexual relationship quickly…he will build another sexual relationship with someone else…I have fear of this... Latifa, woman, mid 30s, 9 years in Australia (interview participant).

Some women explained that they did not engage in sexual activity at their husband’s every request. A few women spoke about directly declining to have sex with their husbands. At times, these participants implied a sense of self-reproach for not being more amenable with their husband’s desires. However, more commonly women explained the ways that they would indirectly evade sexual advances to spare their husbands’ feelings and protect the marital relationship.

I'm not really very interested to have sex…most of the time I don't want to do it…mentally I'm not ready to…If my husband asks me to have [sex] I just say, “No!” Sometimes he's fine, but sometimes he get angry. I'm feeling that he didn't deserve that, cause he's really very good guy. It's just that he wants [to have sex] but I'm not able to do it…I say, “Don't touch me, I don't want!” in a very rude way. And after that when he gets angry I understand that I was so rude and I shouldn't be. Feroza, woman, mid 30s, 12 years in Australia (interview participant).

I’ve never said no to him…because I don't want to hurt his heart, I don’t want him to think I said no to him. If I’m not in mood I pretend I’m sleeping, and he never demanded from me. He always respect and accept. Saera, early 30s, 9 years in Australia (interview participant).

Many men in this study indicated an awareness of the power imbalance between them and their wives, and recognized the overwhelming obligation that women likely feel to have sex. To overcome this issue, some men described that they attempted to observe women’s non-verbal cues and determine their level of interest prior to initiating sexual activity. Several participants explicitly stated that their consideration of women in this area had substantially evolved since living in Australia, and establishing new beliefs that appropriate and enjoyable sexual activity requires both parties to be suitably willing.

In Afghanistan and Iran, if a man wants sex, the wife must do sex with him…But, that culture belongs over there…Here [in Australia] everyone is more understanding with each other. Here it is completely different. Sayed, man, mid 20s, 4 years in Australia (interview participant).

If I say I want to have [sex], it’s no problem, my wife has never said no to me. But sometimes if I look at her, I feel she's sick, or not good, so I never go for sex then, I never ask. I don't want to force my wife, and I know if I ask, she will say yes, but I don't feel good. Abdul, man, early 30s, 6 years in Australia (interview participant).

## Discussion

This community-based participatory research has explored the perspectives and experiences of Afghan women and men living in Australia in relation to sex, sexuality, and intimate relationships. Upon resettlement, Afghan women and men are being exposed to sexual content, cultures, and experiences that are considerably different from their country of origin. This study has provided new insights into the ways that Afghan community members are moving between contrasting societies, and how their understandings of sexual identity, roles, rights, performance, pleasure, desire, health, and consent are being challenged, reshaped, and reconstructed throughout this process. Further, this study has elucidated the transcultural tensions that emerge as Afghan couples navigate unfamiliar territories, and explore new sexual ideas and practices.

Consistent with prior migrant research (Muchoki, 2015), men in this study described having increased access to sex and sexually explicit materials since living in Australia. Within this context, some Afghan men indicated a willingness to transgress traditional cultural values of appropriate sexual behavior by actively seeking and engaging with new sexual cultures, including paying for sex at brothels. These men rationalized their interactions with sex workers by drawing on gendered stereotypes that frame masculine sexual desire as being intrinsic, and, at times, uncontrollable, and women as necessary to fulfil men’s sexual needs (Jordan, 1997; Khoei et al., 2008). This finding contrasts with prior research in which Muslim-background men have vehemently adhered to their traditional cultural and religious beliefs and resisted premarital and extramarital sexual encounters when moving to more sexually liberal societies (Gerholm, 2003).

While Afghan men in Australia initially sought sex outside of marriage to meet their immediate sexual “needs”, they also perceived these sexual interactions to be valuable learning opportunities. For many men, patriarchal norms within Afghanistan appear to have limited their understandings of sex and intimacy, and transcultural sexual encounters were central to their experiences of shifting and reconstructing beliefs and practices around pleasure, gender roles, and power relations. These men described that through these experiences, they had gained a sense of needing to take more responsibility for their wives’ pleasure and sexual needs. That is, they ultimately wanted to apply these new learnings to their marriages in an attempt to establish more egalitarian power dynamics within their intimate relationships. Consistent with prior research, men in the current study were reevaluating cultural and gendered norms around sex that were constraining both their own sexual experiences, and those of their partners (Dworkin et al., 2013; Dworkin & O’Sullivan, 2005; Seal & Ehrhardt, 2003). Within this, men expressed a desire to shift male-dominated sexual initiation patterns, and evolve more respectful, mutually satisfying intimate relationships with their wives (Dworkin & O’Sullivan, 2005).

Within this study, Afghan men’s contending views of sex outside of marriage highlight a unique paradox. On the one hand, male participants appear to be reducing their pre-marital and extra-marital encounters with non-Afghan women as being just for sex; a finding which resonates with existing literature reporting that some migrant men reserve authentic, long-term intimacy for women within their own cultural community (Muchoki, 2015). However, on the other hand, Afghan men are also attributing worth and dignity to these women, because they provide opportunities for them to gain valuable experience around more fulfilling sex lives. For these men, expressing resistance towards dominant cultural meanings of gender and sexual identity provides a way of challenging what it means to be a good husband, and possibly what it means to be a good wife.

This study has also provided new insights into how Afghan women are challenging cultural norms and practices around gender, sex, and desire as they interact with more modernizing values and practices within Australia. Consistent with the experiences of many Afghan men, women described a growing awareness of sexuality. For some female participants this included accessing formal and informal sexual health information—both online, within healthcare settings, and through the media. Although many Afghan women reported that it was their male partners who were initiating and driving conversations and health seeking in this area, some women indicated that they had become more comfortable communicating with their husbands around sex.

However, the majority of women in this study expressed difficulty prioritizing their sexuality, and claiming value and ownership over more pleasurable sexual encounters. For these participants, their female and sexual identity intersected with culturally and religiously constructed notions of sexual modesty and self-respect, which required presenting as sexually innocent, and desirable without desires (Tolman et al., 2003). This was further illustrated by Afghan women’s reluctance to initiate or decline sexual activity with their husbands, which would require departing from traditional gendered sexual norms that are male-dominated and encourage passivity among women (Dworkin & O’Sullivan, 2005). The power-play underpinning this norm, in which women are seeking agency and control, appears to be in contention with the reality that women’s decisions to have sex are primarily based on their partner’s desires, rather than their own. Sexual obedience, duty, and passivity could be viewed as reinforcing positions of subordination for women. However, consistent with prior studies, for many female participants, these seemingly paradoxical and unresolvable relations of power may also be highly valued components of Afghan women’s cultural, religious, gender, and sexual identity (Khoei et al., 2008; Wray et al., 2014).

Consistent with prior research, the current study illuminated the ways that moving to a more sexually liberal culture can open valuable opportunities for women and men to increase their sexual knowledge and awareness, and to reshape understandings, beliefs, meanings, and practices related to sex and intimate relationships (Ahmadi, 2003; Kalra & Bhugra, 2010). By applying the conceptual lens of transculturalism—which moves beyond notions of culture as being unified, fixed and static—this study has highlighted that Afghan cultures in Australia are dynamic and open to change, and this is creating new prospects for enriching sexual and reproductive health for individuals and couples (Kalra & Bhugra, 2010).

However, this research has also illuminated the ways that contrasting and contending ideals and values of identity, gender, sexuality, religion, and culture are interacting to create new challenges for the Afghan community in Australia in regards to sex and relationships. For example, Afghan men intimated sexual awakenings; both in relation to themselves, and for women’s sexual participation, consent, pleasure, and satisfaction. However, in tension with their evolving beliefs and practices, men also expressed fears about women exercising any sexual liberties outside of the home, and, at times, this appeared to place women’s everyday behavior under increased scrutiny. This finding resonates with prior research highlighting men’s experiences of fear and anxiety when they perceive that women’s social status or position of power has shifted (Dworkin et al., 2013, 2015; Hondagneu-Sotelo & Hondagneu-Sotelo, 1994; Withers et al., 2015). Some women also voiced concerns about how easily men can access sex outside of marriage within Australia, and described how this amplified their sense of obligation to be sexually compliant and meet their husband’s desires. These findings highlight how traditional, patriarchal gender roles and expectations can become more entrenched as a result of living within a more liberal society. On these occasions, the intersections, interactions, and negotiations of identity, gender, culture, power, and sexuality are creating hurdles for Afghan individuals and couples as they seek to establish new lifestyles within Australia.

### Practice and Research Implications

This research has provided new insights into Afghan women and men’s experiences of sex, sexuality, and intimate relationships within Australia. A greater understanding of the opportunities and tensions that lie within these can be used to inform how health professionals work alongside Afghan communities, both in Australia and comparable settings.

First, many participants, particularly men, expressed that they valued having access to health professionals to address their sexual health needs, and identified that general practitioners were a highly regarded and trusted source of information. As such, health professionals should be encouraged to ask Afghan women and men questions about their sexual and reproductive health, as these insights highlight that they are often wanting and willing to discuss and address issues in this area. However, findings have also suggested several areas for health professionals to approach with sensitivity. For example, consistent with prior research, the current study demonstrates the importance of understanding how men react to shifting terrain of gendered power relations (Withers et al., 2015). As such, education provided to newly arrived communities on women’s capacity for self-determination in Australia needs to be considerate and inclusive, in order to foster and protect a sense of trust and security between couples, families, and communities. Furthermore, the ways that Afghan women conceptualize and exercise power and control within their sexual negotiations, and the meanings and value they apply to sexual modesty, appear to be closely intertwined with their sense of identity and should therefore be broached with understanding.

This research has also highlighted Afghan women and men’s considerable agency to challenge social conventions and effect change. In particular, the paradoxes presented provide insights into footholds for social change driven by Afghan people themselves, rather than top-down sexual and reproductive health workers. Although largely overlooked in contemporary sexual and reproductive health promotion, this study suggests that for some Afghan women and men, transgressing traditional cultural practices and beliefs may create alternative possibilities for them to build healthy, respectful, and fulfilling sex lives and intimate relationships as they settle within Australia.

Lastly, this study highlights the value of participatory approaches that open up safe, discursive space for Afghan individuals to voice their sexual and reproductive health experiences. Ongoing and productive discussions with Afghan women and men are important to acknowledge the shifting meanings of sex and intimacy in their everyday lives, and to ensure that responses are based on current need and driven by the community themselves.

### Strengths and Limitations

The two-phased, qualitative approach employed in this research was well suited to the exploratory, cross-cultural, and sensitive nature of the study. Conducting the focus group discussions prior to the semi-structured interviews enabled broad preliminary themes to be established in a group context, which could then be explored, in-depth, within a more private setting. The collaborative partnership with members of the Afghan community is a further strength of this research. In addition to overcoming a range of logistical challenges, this approach enhanced cultural competency throughout the research process, and enabled the sexual experiences of Afghan people to be thoroughly explored with sensitivity. In addition, Afghanistan-born people, particularly adult, married, women and men, have been noticeably underrepresented in prior research (that is available in English) focusing on sexuality, both within Australia and internationally.

There are several limitations to this study. First, bias is an inherent risk within purposive sampling. To mitigate this, participant recruitment was guided by a clearly defined inclusion and exclusion criteria, and a maximum variation sampling framework was applied to ensure representation of participants with a range of characteristics, attitudes, beliefs, and experiences. Nevertheless, it is possible that people with more traditional views or who are more private about their sexual experiences were either not reached, or declined to participate. Further to this, due to the sensitive, and traditionally culturally taboo nature of this subject area, it is likely that some participants were more guarded and reserved than others. In line with purposive sampling, researchers intentionally recruited people who were available, willing, and able to share their lived-experiences, and employed appropriate methods to ensure individuals felt safe and comfortable throughout recruitment, data collection and follow-up. Regardless, the authentic stories of participants may not have been captured in equal degrees of depth and detail. Therefore, the perspectives and experiences presented in this paper may not be representative of the wider Afghanistan-born population living in Australia, including people who are not engaged with the health and social care system, or community groups and organizations.

Lastly, the majority of the data required translation from Dari into English. Extensive precautions were taken to maintain data accuracy and quality; however, this still contributed an additional layer of interpretation between the raw data and the findings.

### Conclusion

This research has provided insights into the ways that interacting social and cultural contexts influence sex, sexuality, and intimate relationships. These findings emphasize the importance of recognizing the fluid and dynamic nature of sexual and reproductive health understandings, values, and beliefs. For Afghans in Australia, migrating to a more sexually liberal country presents valuable opportunities to embrace new ideas and practices within this area. However, participants’ experiences were double-edged, as transcultural tensions also emerged through contending perspectives and needs that occurred at the intersections of gender, power, religion, and culture. The paradoxes, trade-offs and areas of ambiguity that Afghan women and men experience around gender roles, sex, and intimate relationships as they transition to life in Australia constitute a significant gap in the published research. By understanding these tensions as footholds for change, rather than cultural barriers, health professionals can better work together with refugee and migrant communities to promote positive and respectful sexuality and relationships in the contexts of their transcultural lives.

## Footnotes

1 The population of Afghanistan is comprised of multiple ethnic groups. However, after consultation with Afghanistan-born community researchers, throughout this paper all people born in Afghanistan will be referred to as “Afghan.”
